# Leucine Carboxyl Methyltransferase Downregulation and Protein Phosphatase Methylesterase Upregulation Contribute Toward the Inhibition of Protein Phosphatase 2A by α-Synuclein

**DOI:** 10.3389/fnagi.2018.00173

**Published:** 2018-06-08

**Authors:** Hao Tian, Yongquan Lu, Jia Liu, Weijin Liu, Lingling Lu, Chunli Duan, Ge Gao, Hui Yang

**Affiliations:** Department of Neurobiology Capital Medical University, Center of Parkinson’s Disease Beijing Institute for Brain Disorders, Beijing Key Laboratory on Parkinson’s Disease, Key Laboratory for Neurodegenerative Disease of the Ministry of Education, Beijing Center of Neural Regeneration and Repair, Beijing, China

**Keywords:** Parkinson’s disease, α-synuclein phosphorylation, PP2A methylation, LCMT-1, PME-1, PP2A regulatory subunits

## Abstract

The pathology of Parkinson’s disease (PD) is characterized by intracellular neurofibrillary tangles of phosphorylated α-synuclein (α-syn). Protein phosphatase 2A (PP2A) is responsible for α-syn dephosphorylation. Previous work has demonstrated that α-syn can regulate PP2A activity. However, the mechanisms underlying α-syn regulation of PP2A activity are not well understood. In this study, we found that α-syn overexpression induced increased α-syn phosphorylation at serine 129 (Ser129), and PP2A inhibition, *in vitro* and *in vivo*. α-syn overexpression resulted in PP2A demethylation. This demethylation was mediated via downregulated leucine carboxyl methyltransferase (LCMT-1) expression, and upregulated protein phosphatase methylesterase (PME-1) expression. Furthermore, LCMT-1 overexpression, or PME-1 inhibition, reversed α-syn-induced increases in α-syn phosphorylation and apoptosis. In addition to post-translational modifications of the catalytic subunit, regulatory subunits are involved in the regulation of PP2A activity. We found that the levels of regulatory subunits which belong to the *PPP2R2* subfamily, not the *PPP2R5* subfamily, were downregulated in the examined brain regions of transgenic mice. Our work identifies a novel mechanism to explain how α-syn regulates PP2A activity, and provides the optimization of PP2A methylation as a new target for PD treatment.

## Introduction

Parkinson’s disease (PD) is a progressive neurodegenerative disorder. It is characterized by the death of dopaminergic neurons within substantia nigra, and the development of phosphorylated α-synuclein (α-syn) tangles.

α-syn is a PD-related protein, encoded by the *SNCA* gene. α-syn is highly expressed in the human brain where it regulates synaptic plasticity, dopamine release, and lipid metabolism(Golovko et al., 2009; Gao et al., 2015; Bellucci et al., 2016). *SNCA* duplication and triplication is associated with an increased risk of developing PD (Farrer et al., 2004). Phosphorylation at serine 129 (Ser129) is one of the most important post-translational modifications at α-syn. Approximately 4% of α-syn in the normal human brain is phosphorylated at Ser129. However, a significant accumulation of phosphorylated α-syn at Ser129 (>90%) has been found within Lewy bodies of PD brains (Fujiwara et al., 2002; Anderson et al., 2006). Levels of phosphorylated α-syn are also positively correlated with disease severity, suggesting that this post-translational modification participates in the regulation of disease progression (Beach et al., 2009; Zhou et al., 2011; Zapiec et al., 2017).

Protein phosphatase 2A (PP2A), a major serine-threonine phosphatase in the brain, regulates numerous cell signaling cascades by impeding the activity of kinase genes (Strack et al., 1997). PP2A comprises a wide range of regulatory subunits which determine substrate specificity, as well as a core enzyme containing scaffold A and catalytic C subunits (Slupe et al., 2011). PP2A activity is regulated by the phosphorylation, as well as the methylation, of the PP2A catalytic subunit. In our previous study, we found that α-syn could inhibit PP2A activity by increasing Src-mediated PP2A phosphorylation (Yang et al., 2013). Additionally, PP2A methylation, which is regulated by Leucine carboxyl methyltransferase (LCMT-1) and PP2A methyltransferase (PME-1), is of great interest, due to its association with PP2A activity, as well as its role in recruiting specific substrates (Lee and Stock, 1993; Ogris et al., 1999). In the development of tau pathology in Alzheimer disease (AD), decreases in PP2A methylation correlate with disease severity (Sontag et al., 2004). The significance of PP2A methylation is further highlighted through the discovery that reduced PP2A methylation, reduced LCMT-1, and elevated PME-1 expression have been found in postmortem brains of PD and AD patients (Park et al., 2016, 2017). Furthermore, PP2A regulatory subunits play an important role in determining catalytic activity. As for specific regulatory subunits, the *PPP2R2A* subunit is a major phosphatase which dephosphorylates phosphorylated α-syn (Lee et al., 2011). The *PPP2R5* subunits show decreased activity toward phosphorylated tau, when compared with the *PPP2R2* subunits (Xu et al., 2008). PP2A methylation has been shown to be essential in recruiting regulatory subunits that belong to the *PPP2R2* subfamily, whilst *PPP2R5* subunits are not affected (Longin et al., 2007). The findings to date suggest that PP2A methylation and PP2A regulatory subunits are involved in PP2A inactivation associated with neurodegenerative diseases. Several studies have reported that α-syn, a key protein in PD pathology, can regulate PP2A activity (Peng et al., 2005; Lou et al., 2010; Wu et al., 2012; Liu et al., 2015). However, the mechanisms underlying how α-syn regulates PP2A activity remain poorly understood.

In this study, we investigated the mechanism underlying α-syn regulation of PP2A activity during α-syn-induced increases in α-syn phosphorylation. We found that α-syn overexpression increased PP2A demethylation at Leu309, resulting in PP2A inhibition. We also found that this PP2A demethylation was accompanied by downregulated LCMT-1, and upregulated PME-1 expression. Furthermore, LCMT-1 overexpression or PME-1 inhibition alleviated α-syn-induced cell injury. *PPP2R2* subfamily regulatory subunit levels were also downregulated in the examined brain regions of transgenic (Tg) mice. Our findings suggest that α-syn negatively regulates PP2A methylation via LCMT-1 and PME-1, whilst activation of LCMT-1 and inhibition of PME-1 reverses this process. These results help to elucidate the mechanisms underlying how α-syn regulates PP2A.

## Materials and Methods

### Reagents

Both protease and phosphatase inhibitors were purchased from Roche (Basel, Switzerland). Primary antibodies used in western blotting are as follows: Mouse anti-β-actin (1:5000, Proteintech), Rabbit anti-p-α-syn (1:1000, Abcam), Mouse anti-p-α-syn (1:1000, Wako), Mouse anti-α-syn (1:1000, Santa Cruz), Rabbit anti-Human α-syn (1:2000, Abcam), Mouse anti-demethylate-PP2A (1:1000, Millipore), Mouse anti-PP2A (1:1000, BD biosciences), Mouse anti-β-tubulin (1:1000, Abcam), Mouse anti-*PPP2R2A* (1:1000, CST), Mouse anti-*PPP2R2B* (1:1000, Abcam), Rabbit anti-*PPP2R2D* (1:1000, Abcam), Mouse anti-*PPP2R5A* (1:500, Santa Cruz), Rabbit anti-*PPP2R5D* (1:5000, Abcam), Rabbit anti-*PPP2R5E* (1:1000, Abcam), Mouse anti-LCMT-1 (1:1000, Abcam), Rabbit anti-PME-1 (1:1000, Millipore), Mouse anti-GAPDH (1:3000, Sigma), Mouse anti-c-Myc (1:5000, Clontech), and Mouse anti-FLAG (1:500, Santa Cruz). Secondary antibodies are as follows: Goat anti-Rabbit IRdye680 (LI-COR Bioscience), Goat anti-Mouse IRdye680 (LI-COR Bioscience), Goat anti-Rabbit IRdye800 (LI-COR Bioscience), Goat anti-Mouse IRdye800 (LI-COR Bioscience), Goat anti-Rabbit Alexa Fluor 488 (Thermo Fisher Scientific). The inhibitor of protein phosphatase methylesterase-1 (PME-1), AMZ30, was purchased from MedChem Express (Shanghai, China). Dulbecco’s Modified Eagle’s Medium (DMEM), and fetal bovine serum (FBS) were purchased from Gibco.

### Cell Culture and Transfection

WT human α-syn cDNA was cloned into pCMV-Myc and pmCherry-N_1_ vectors. Plasmids encoding pcDNA3.1-3 × Flag-HLCMT-1 were purchased from Youbio (Changsha, China). SK-N-SH cells obtained from the American Type Culture Collection (ATCC) were maintained in DMEM plus 10% FBS and penicillin/streptomycin (100 U/ml). Prior to transfection, the medium was changed to Opti-MEM, and the required amounts of plasmid and Lipofectamine 2000 were added.

### Primary Cortical Neurons Culture and Infection

Lentiviral gene transfer vectors were purchased from Genechem (Shanghai, China). shRNA to knockdown endogenous rat α-syn and scrambled sequences are listed as follows: LV-sh-α-syn, 5′-GCA CAC TGT TCC TCG TTA TGA-3′; LV-sh-con, 5′-GGA TTG ATT CAA CAC GGA AGA-3′. Primary cortical neuronal cultures were prepared from embryonic day 14 Sprague-Dawley rats or C57BL/6 mice, respectively, as previously described (Du et al., 2015). Neurons were plated in FBS-supplemented DMEM medium. After 4 h, the medium was changed to Neurobasal/B27 medium. At 7 days *in vitro* (DIV), mouse neurons were harvested and rat neurons were infected with the aforementioned lentiviruses.

### Animals

All procedures were approved by the Animal Care and Use Committee of the Capital Medical University. Animal experiments were performed in accordance with the National Institutes of Health (NIH) Guide for laboratory animals. Thy-1 α-syn Tg mice, which express human α-syn, and α-syn KO mice were, respectively, purchased from the Jackson Laboratory (Bar Harbor, ME, United States) and Model Animal Research Center of Nanjing University (Nanjing, China). Mice were maintained on a C57BL/6N background. All mice were housed on a daily 12 h light/dark cycle. Mouse genotype was identified by polymerase chain reaction using genomic DNA from tail biopsies. Nine-month-old male mice were used for this study, and brain tissue was isolated and then stored at -80°C.

### Sample Preparation and Western Blotting

Brain tissue and cell homogenates were prepared in radio immunoprecipitation buffer (RIPA) buffer, according to previously described methods (Yang et al., 2013). After centrifugation, the supernatant fraction was collected and termed RIPA soluble fraction. The pellet was resuspended in 8 M urea/8% SDS and termed the RIPA insoluble fraction. Protein content was measured using a bicinchoninic acid (BCA) assay, purchased from Pierce. Proteins (30 μg) were separated on 12% sodium dodecyl sulfate polyacrylamide gel electrophoresis (SDS/PAGE) and transferred onto a polyvinylidene fluoride (PVDF) membrane followed by blocking in 10% skimmed milk. Membranes were then incubated with the primary antibodies overnight. Secondary antibodies were incubated for 1 h. Western blots were imaged using a Li-cor IR Odyssey imaging system (NE, United States). Image J software (NIH, Bethesda, MD, United States) was used to measure band intensity.

### Immunofluorescence

For immunocytochemistry, cells transfected with pmCherry-N_1_ or pmCherry-N_1_-α-syn were fixed with 4% (wt/vol) PFA. The cells were then permeabilized with 0.3% Triton X-100. For immunohistochemistry, mouse brains were fixed with 4% PFA and sunk in 30% sucrose. Brains from Wt and α-syn Tg mice were cut to generate 30 μm thick coronal sections. Proteinase-K (PK) treatment (20 μg/ml in PBST buffer at 37°C for 20 min) was applied to Wt and Tg mouse brain slices. After blocking with goat serum, anti-p-α-syn (1:1000, Wako), anti-Human-α-syn (1:1000, Abcam), and secondary antibodies were used for immunofluorescence. Cells were treated with 4′,6-diamidino-2-phenylindole (DAPI) (1:10000, Invitrogen) for 10 minutes. Images were acquired on a Leica SP8 microscope (Leica LAS software).

### PP2A Activity Assay

PP2A activity was detected using a PP2A activity assay (GMS50042.3, GENMED) as previously described (Yang et al., 2013). Briefly, cell extracts and homogenates from different brain regions of α-syn Tg mice and controls were prepared in lysis buffer provided in the kit. The ability to dephosphorylate the phosphopeptide K-R-pT-I-R-R was used to determine PP2A activity in the cell and brain samples.

### Co-IP

Cells extracts (800 μg) from the different groups or brain homogenates (800 μg) from the cortex of Wt and Tg mice were used for Co-Immunoprecipitation as described previously (Yang et al., 2013). Protein lysate was incubated with 3 μg antibody at 4°C for 3 h and then 30 μL Protein G-Sepharose beads were added into the mixture. After incubation for 12 h, the beads were collected and resuspended with loading buffer for western blotting.

### Annexin V FITC Assay

The Annexin V-FITC apoptosis detection kit (Merck Millipore) was used to determine the cell apoptosis. SK-N-SH cells were plated in 24-well plate and then collected after transfection or treatment with AMZ30. Annexin V-FITC was added into the cell suspension and incubated at room temperature for 15 min. Propidium Iodide was added followed by flow cytometry analysis.

### Cell Viability

The CCK-8 was purchased from MedChem Express, and was used to assess cell viability. SK-N-SH cells were plated in 96-well plates. Transient expression of indicated plasmids or treatment with AMZ30 was performed. CCK-8 solution was then added to the medium and incubated for 2 h at 37°C in an incubator. Optical density (OD) was determined using a microplate reader at 450 nm for each group.

### Statistical Analysis

Data are expressed as mean ± SD, and GraphPad Prism 5 (GraphPad Software Company, CA, United States) was used for the analyses. The evaluation of normality was examined by the Kolmogorov–Smirnov (KS) test. Comparisons were performed using analysis of variance (ANOVA) followed by Bonferroni’s *post hoc* test for three groups or more, and Student’s *t*-test for two groups. The homogeneity of variance prior to Student’s *t*-test or ANOVA was examined individually by the *F*-test or the Brown–Forsythe test. *P* value < 0.05 indicates a statistically significant difference.

## Results

### α-Syn Overexpression Induced Increases in α-Syn Phosphorylation at Ser129 via PP2A Inactivation

**FIGURE 1:**
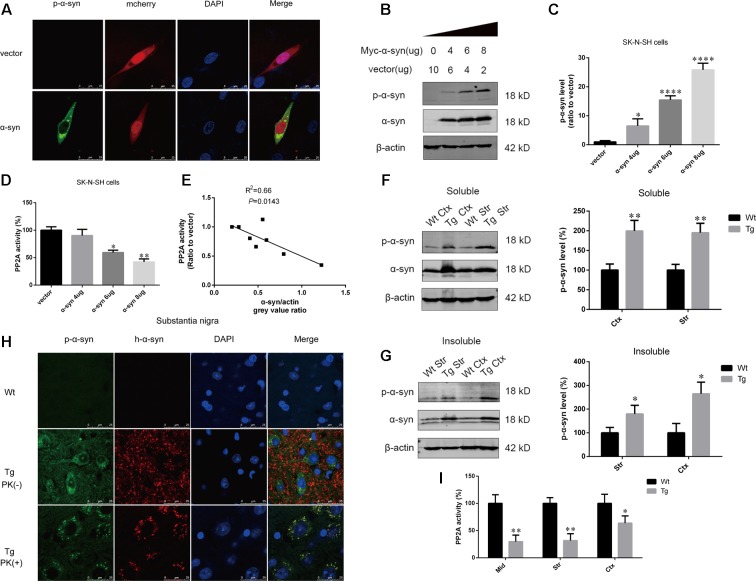
α-syn overexpression increased α-syn phosphorylation at Ser129. **(A)** Representative immunofluorescence images showing cells transfected with pmCherry-N_1_ or pmCherry-N_1_-α-syn stained with the anti-phosphorylated α-syn antibody. Scale bar = 25 μm. **(B,C)** Immunoblot results and quantifications of phosphorylated α-syn at Ser129 (p-α-syn) and α-syn levels in SK-N-SH cells transfected with increasing amounts of pCMV-Myc-α-syn (4, 6, and 8 μg). pCMV-Myc plasmid was added to keep the total amount of DNA equal in each transfection. β-actin was used as a loading control. The ratio of p-α-syn to β-actin in vector group was considered as 100%. **(D)** Measurement of PP2A activity for the aforementioned groups. **(E)** Correlation between α-syn level and PP2A activity. **(F,G)** Immunoblot results and quantifications of p-α-syn and total α-syn level in the RIPA soluble and insoluble fraction of cortex as well as striatum of α-syn Wt and Tg mice. β-actin was used as a loading control. The ratio of p-α-syn to β-actin in Wt group was considered as 100%. **(H)** Representative immunofluorescence images from Wt and Tg mice slices of substantia nigra treated with or without PK. **(I)** Measurement of PP2A activity in examined brain regions of Wt and Tg mice. Data are expressed as mean ± SD. Kolmogorov–Smirnov (KS) test; *P* > 0.05. In **C,D**: Brown–Forsythe test; *P* > 0.05; one-way analysis of variance; ^∗^*P* < 0.05, ^∗∗^*P* < 0.01, ^∗∗∗∗^*P* < 0.0001 vs. vector (*n* = 3); In **F,G,I**: F-test; *P* > 0.05; unpaired Student’s *t*-test; ^∗^*P* < 0.05, ^∗∗^*P* < 0.01 vs. Wt mice (*n* = 3). The associations between α-syn level and PP2A activity were determined with Pearson correlation analysis.

To determine whether α-syn overexpression leads to increased α-syn phosphorylation at Ser129, we performed immunofluorescence in SK-N-SH cells transfected with pmCherry-N_1_ or pmCherry-N_1_-α-syn, which were stained with an anti-phosphorylated α-syn antibody. Phosphorylated α-syn at Ser129 was present in the α-syn transfected group. Phosphorylated α-syn has been reported to easily form aggregates (Fujiwara et al., 2002). Intracelluar inclusions, containing phosphorylated α-syn, were observed in cells transfected with α-syn (**Figure 1A**). For further confirmation, increasing amounts of pCMV-Myc-α-syn were overexpressed in SK-N-SH cells. We observed that α-syn induced a dose-dependent increase in α-syn phosphorylation at Ser129 and decrease in cell viability, after transfection for 24 h (**Figures 1B,C** and Supplementary Figure S1). PP2A is the major phosphatase which controls α-syn dephosphorylation. To determine whether PP2A was regulated in this process, we measured PP2A activity at each α-syn level and performed correlational analysis examining the relationship between PP2A activity and α-syn levels. PP2A activity decreased as α-syn level increased (**Figure 1D**). A moderate correlation was observed between PP2A activity and α-syn expression, with an *R*^2^ value of 0.66 (**Figure 1E**). To examine whether overexpression of exogenous α-syn could increase α-syn phosphorylation at Ser129 *in vivo*, the expression of phosphorylated α-syn at Ser129 and total α-syn in the RIPA soluble and insoluble fraction of cortex and striatum of α-syn Tg mice and their Wt littermates were detected. Increases in phosphorylated α-syn at Ser129, and PP2A inactivation were found in the aforementioned brain regions of α-syn Tg mice when compared with controls (**Figures 1F,G,I**). Human α-syn and phosphorylated α-syn were only seen in the substantia nigra of Tg mice. Furthermore, they were both partly PK-resistant and PK-resistant α-syn was distributed in neuronal cytoplasm with dot-like structures (**Figure 1H**). These results suggest that α-syn overexpression can lead to PP2A inactivation, which contributes to increased α-syn phosphorylation and aggregation.

### α-Syn Regulated PP2A Demethylation at Leu309 *in Vitro* and *in Vivo*

**FIGURE 2:**
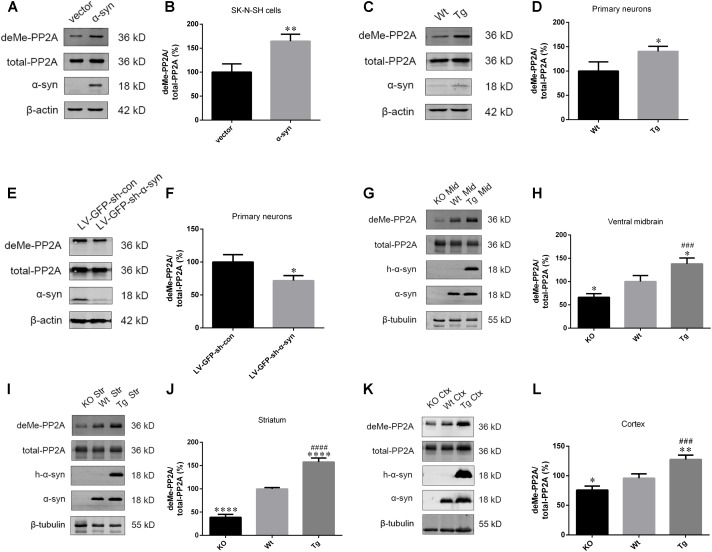
α-syn regulated PP2A demethylation at Leu309 *in vitro* and *in vivo*. **(A,B)** Immunoblot results and quantifications of demethylated PP2A (deMe-PP2A), total-PP2A, and α-syn levels in SK-*N*-SH cells transfected with pCMV-Myc or pCMV-Myc-α-syn for 24 h. The ratio of deMe-PP2A to total-PP2A in vector group was considered as 100%. **(C,D)** Immunoblot results and quantifications of deMe-PP2A, total-PP2A, and α-syn in Wt and Tg neuron lysates. The ratio of deMe-PP2A to total-PP2A in Wt group was considered as 100%. **(E,F)** Immunoblot results and quantifications of deMe-PP2A, total-PP2A, and α-syn in rat primary neurons infected with LV-GFP-sh-con or LV-GFP-sh-α-syn vectors. The ratio of deMe-PP2A to total-PP2A in LV-GFP-sh-con group was considered as 100%. **(G–L)** Immunoblot results and quantifications of deMe-PP2A, total-PP2A, human α-syn (h-α-syn) and α-syn in brain homogenates from α-syn KO, Wt and α-syn Tg mice. β-tubulin was used as a loading control. The ratio of deMe-PP2A to total-PP2A in Wt group was considered as 100%. Data are expressed as mean ± SD. KS test; *P* > 0.05; In **A–F**: *F*-test; *P* > 0.05; unpaired Student’s *t*-test; ^∗^*P* < 0.05 vs. vector or LV-GFP-sh-con (*n* = 3); In **G–L**: Brown–Forsythe test; *P* > 0.05; one-way analysis of variance; ^∗^*P* < 0.05, ^∗∗^*P* < 0.01, ^∗∗∗∗^*P* < 0.0001 vs. Wt mice (*n* = 3); ^###^*P* < 0.001, ^####^*P* < 0.0001 vs. KO mice (*n* = 3).

Based on our finding that α-syn overexpression causes PP2A inhibition through increasing PP2A phosphorylation, and given that PP2A methylation is also involved in the regulation PP2A activity (Supplementary Figures S2–S4), the impact of α-syn on PP2A methylation was investigated. We found that α-syn overexpression resulted in PP2A demethylation at Leu309 in SK-N-SH cells (**Figures 2A,B**). Similarly, primary cortical neurons from Tg mice showed significantly more demethylation at PP2A (**Figures 2C,D**). On the other hand, the level of PP2A demethylation substantially decreased in rat primary cortical neurons with endogenous α-syn knock-down (**Figures 2E,F**). Similar to the *in vitro* results, elevated PP2A demethylation was found in the examined brain regions of Tg mice when compared with Wt mice of the same age. We further detected the expression of demethylated PP2A in the aforementioned brain regions of α-syn knockout (KO) mice. Reduced PP2A demethylation was found in the KO mice compared to wild type (Wt) mice. No α-syn was detected in the KO mice brains (**Figures 2G–L**). These data suggest that PP2A methylation is also involved in PP2A inhibition by α-syn, and not just by PP2A phosphorylation.

### α-Syn Overexpression Downregulates LCMT-1 Whilst Upregulating PME-1, Whereas α-Syn Knockout Demonstrated the Opposite Effects

**FIGURE 3:**
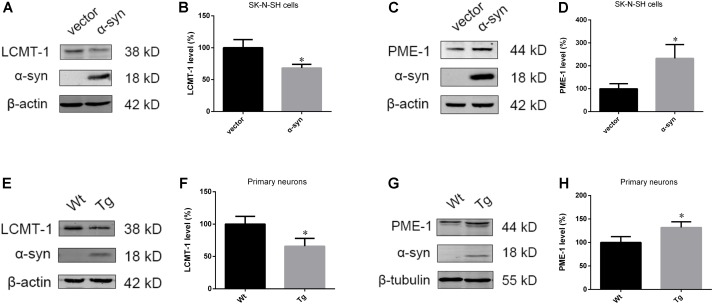
α-syn overexpression downregulated LCMT-1 while upregulating PME-1 *in vitro*. **(A–D)** The expression of LCMT-1 and PME-1 was determined with western blotting in cells transfected with pCMV-Myc or pCMV-Myc-α-syn. β-actin was used as a loading control. The ratio of LCMT-1 or PME-1 expression to β-actin in vector group was considered as 100%. **(E–H)** Western blot analysis of LCMT-1, PME-1 and α-syn in α-syn-overexpressing neurons and controls. β-actin and β-tubulin were used as loading controls. The ratio of LCMT-1/β-actin or PME-1/β-tubulin in vector group was considered as 100%. Data are expressed as mean ± SD. KS test; *P* > 0.05; *F*-test; *P* > 0.05; unpaired Student’s *t*-test; ^∗^*P* < 0.05 vs. vector or Wt primary neurons (*n* = 3).

**FIGURE 4:**
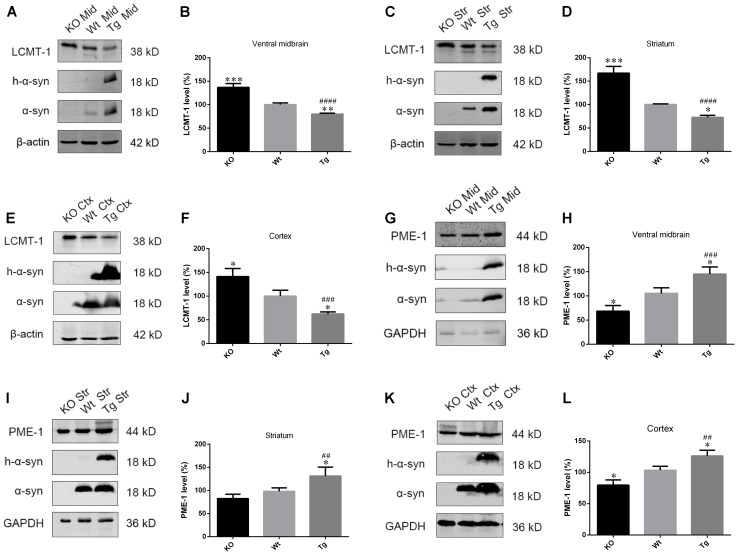
α-syn regulated LCMT-1 and PME-1 expression *in vivo*. **(A–F)** LCMT-1 expression was determined with western blotting in brain homogenates from examined brain regions of the α-syn KO, Wt, and α-syn Tg mice. β-actin was used as a loading control. The ratio of LCMT-1 expression to β-actin in Wt group was considered as 100%. **(G–L)** PME-1 expression was determined with western blotting in brain homogenates from examined brain regions of the α-syn KO, Wt, and α-syn Tg mice. GAPDH was used as a loading control. The ratio of PME-1 expression to GAPDH in Wt group was considered as 100%. Data are expressed as mean ± SD. KS test; *P* > 0.05; Brown–Forsythe test; *P* > 0.05; one-way analysis of variance; ^∗^*P* < 0.05, ^∗∗^*P* < 0.01, ^∗∗∗^*P* < 0.01 vs. Wt mice with the same age (*n* = 3); ^##^*P* < 0.01, ^###^*P* < 0.001, ^####^*P* < 0.0001 vs. KO mice with the same age (*n* = 3).

As PP2A demethylation can be modulated by LCMT-1 and PME-1, we investigated the expression of these proteins. PME-1 levels were significantly increased, and LCMT-1 levels were significantly decreased in α-syn transfected SK-N-SH cells (**Figures 3A–D**). Similarly, primary cortical neurons from Tg mice demonstrated higher levels of PME-1 and lower levels of LCMT-1 (**Figures 3E–H**). To determine whether α-syn overexpression could regulate LCMT-1 and PME-1 expression *in vivo*, we measured homogenates from several brain regions of the Wt, Tg, and KO mice. Remarkably, LCMT-1 levels were reduced and PME-1 levels were increased in the examined brain regions of Tg mice. Furthermore, a reduction in PME-1, and a significant increase in LCMT-1 was observed in α-syn KO mice when compared with Wt mice of the same age (**Figures 4A–L**). These findings indicate that α-syn can regulate the expression of LCMT-1 and PME-1, which are involved in α-syn-induced increases in PP2A demethylation.

### LCMT-1 Overexpression or PME-1 Inhibition Protects Against α-Syn-Induced PP2A Inhibition, and Increases in Apoptosis of SK-N-SH Cells

**FIGURE 5:**
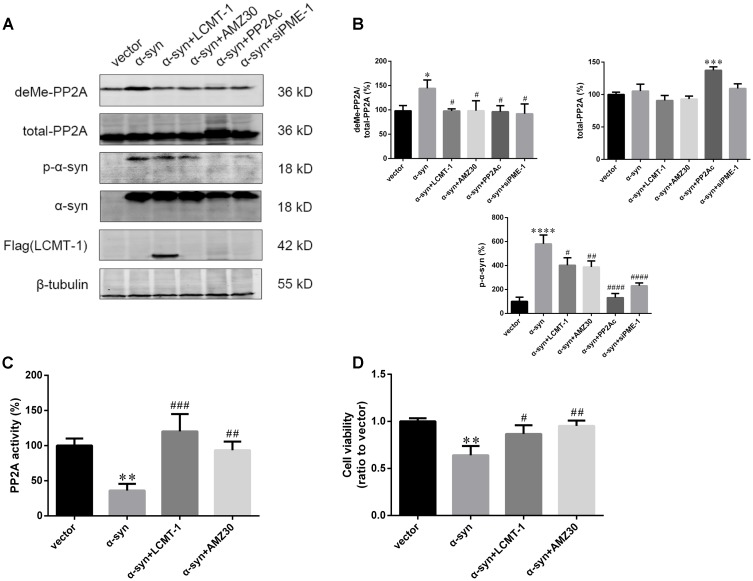
LCMT-1 overexpression or PME-1 inhibition by AMZ30 protected against PP2A inactivation and cell apoptosis mediated by α-syn. **(A,B)** Immunoblotting and quantifications of deMe-PP2A, total-PP2A, *p*-α-syn, α-syn, and Flag-LCMT-1 in vector, α-syn, α-syn + LCMT-1, α-syn + AMZ30, α-syn + PP2Ac, and α-syn + siPME-1 group. β-tubulin was used as a loading control. The ratio of deMe-PP2A to total-PP2A, total-PP2A to β-tubulin, *p*-α-syn to β-tubulin in vector group was individually considered as 100%. **(C)** Histogram showing the PP2A activity in the aforementioned groups. **(D)** Cell viability was measured with CCK-8. Data are expressed as mean ± SD. KS test; *P* > 0.05; Brown–Forsythe test; *P* > 0.05; one-way analysis of variance; ^∗^*P* < 0.05, ^∗∗^*P* < 0.01, ^∗∗∗^*P* < 0.01, ^∗∗∗∗^*P* < 0.0001 vs. vector (*n* = 3); ^#^*P* < 0.05, ^##^*P* < 0.01, ^###^*P* < 0.001, ^####^*P* < 0.0001 vs. α-syn (*n* = 3).

The role of LCMT-1 and PME-1 were further investigated in the case of α-syn-induced increases in PP2A demethylation *in vitro*. This was achieved by LCMT-1 overexpression or incubation with the PME-1 inhibitor, AMZ30 (10 μM for 24 h), following the transfection of α-syn. LCMT-1 overexpression protected against α-syn-induced increases in PP2A demethylation at Leu309, and decreased PP2A activity, whilst no change was observed in total PP2A expression in SK-N-SH cells. Similarly, increased PP2A demethylation and reduced PP2A activity were reversed by treatment with the PME-1 inhibitor AMZ30. This protective effect was also verified by co-transfection of PP2A catalytic subunit (PP2Ac) or PME-1 knockdown through siRNA. These results confirm that α-syn-induced increases in PP2A demethylation occur via LCMT-1 and PME-1. Furthermore, LCMT-1 overexpression or AMZ30 treatment attenuated this increase in phosphorylated α-syn. PP2Ac overexpression and PME-1 knockdown group also showed decreased phosphorylated α-syn level compared with vector group (**Figures 5A–C** and Supplementary Figure S5). As α-syn phosphorylation at Ser129 is involved in α-syn-induced increases in apoptosis, we assessed the extent of apoptosis in the aforementioned groups using the Cell Counting Kit-8 (CCK-8) and Annexin V FITC assay followed by flow cytometry analysis. Treatment with AMZ30 or LCMT-1 overexpression reduced cell apoptosis induced by α-syn, as evidenced by increased cell viability and decreased levels of annexin V-positive cells (**Figures 5D** and Supplementary Figures S6, S7).

### The Expression of Regulatory Subunits Which Belong to the *PPP2R2* Subfamily, Not *PPP2R5* Subfamily, Were Downregulated in the Ventral Midbrain and Cortex of Tg Mice

**FIGURE 6:**
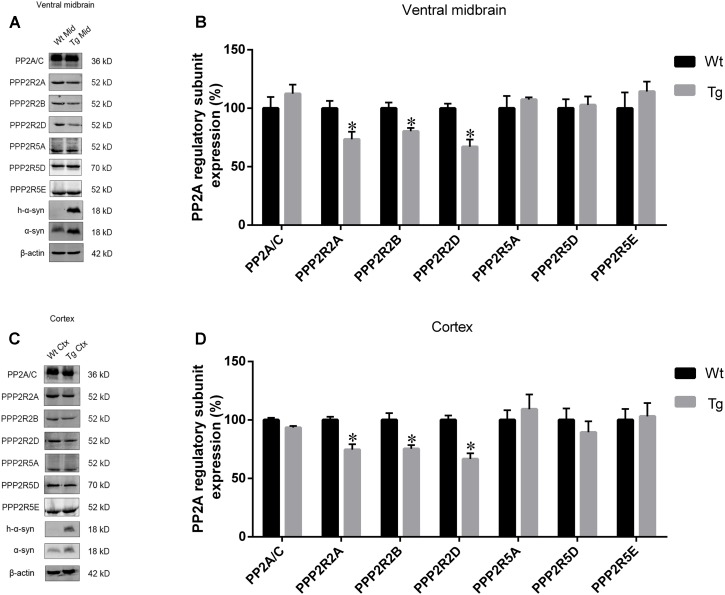
The expression of regulatory subunits which belong to the *PPP2R2* subfamily, not *PPP2R5* subfamily, were downregulated in examined brain regions of Tg mice. **(A,C)** western blot results for PP2A subunit expression from brain homogenates of ventral midbrain as well as cortex of Tg mice and Wt littermates. β-actin was used as a loading control. **(B,D)** quantitative analysis of western blot data. The ratio of individual PP2A subunit expression to β-actin in Wt group was considered as 100%. Data are expressed as mean ± SD. KS test; *P* > 0.05; *F*-test; *P* > 0.05; unpaired Student’s *t*-test; ^∗^*P* < 0.05 vs. Wt littermates (*n* = 3).

As PP2A methylation affects the binding of its catalytic subunit to regulatory subunits, and leads to an increase in regulatory subunit degradation, we analyzed the amount of *PPP2R2* and *PPP2R5* subfamilies in the midbrain and cortex of Tg mice and Wt littermates. α-syn overexpression decreased the expression of *PPP2R2* subfamilies, whilst *PPP2R5* subfamilies were not affected (**Figures 6A–D**). Reduced PP2A methylation and PP2A heterotrimeric enzymes containing *PPP2R2* regulatory subunits led to decreased PP2A activity toward phosphorylated α-syn. These data provided further evidence that α-syn increases PP2A demethylation.

## Discussion

Accumulation and aggregation of phosphorylated α-syn at Ser129 is considered one of the hallmarks of a pathogenic cascade that eventually leads to the development of PD (Braak et al., 2003; Anderson et al., 2006). α-syn phosphorylated at Ser129 has also been observed in grafted dopaminergic neurons of PD brains, and in preformed α-syn fibrils (PFFs)-treated neurons, suggesting its association with PD pathology (Li et al., 2008; Luk et al., 2009). Overexpression of Ser129A α-syn, which cannot be phosphorylated at Ser129, decreases cytoplasmic inclusion formation in cultured cells when compared with coexpression of synphlilin-1 and wild type synuclein (Smith et al., 2005). PP2A is the major phosphate controlling phosphorylated α-syn dephosphorylation. Prior work has demonstrated that PP2A inhibition participates in α-syn- and rotenone-induced α-syn phosphorylation at Ser129 (Yang et al., 2013; Wang et al., 2016). PP2A inactivation by age-dependent α-syn oligomerization is also present in aging monkey brains (Chen et al., 2016). Our study demonstrates that α-syn induces dose-dependent decreases in PP2A activity, thereby resulting in increases in α-syn phosphorylation.

PP2A activity is regulated by its posttranslational modifications included phosphorylation at Tyr307 and methylation at Leu309. In our previous studies, we reported that α-syn overexpression increases PP2A phosphorylation via formation of calmodulin/Src complex. Although PP2A inhibition was reversed by PP2A activator C2-ceramide, the pathogenicity included increased α-syn phosphorylation was not fully abolished. PP2A methylation affects both catalytic activity and the association with regulatory subunits which determine substrate recognition, while phosphorylation reduces its catalytic activity. Thus, we investigated whether PP2A methylation was involved in PP2A inactivation induced by α-syn overexpression. Decreased PP2A methylation and activity has been observed in several neurodegenerative diseases, including AD. Recently, LCMT-1 protein and PP2A methylation levels were found to be decreased in PD and DLB brains, whilst PME-1 protein and PP2A demethylation levels increased (Park et al., 2016). An earlier study also reported that augmented PP2A methylation by EHT protects against α-synucleinopathy and behavioral deficits in α-syn Tg mice (Lee et al., 2011). However, the primary events that underlie α-syn-induced PP2A inhibition has not been fully elucidated. Our study provides compelling evidence that α-syn promotes PP2A demethylation by simultaneously downregulating LCMT-1 expression and upregulating PME-1 expression, resulting in PP2A inhibition. Moreover, demethylated PP2A was reduced in the examined brain regions of α-syn KO mice, while LCMT-1 increased and PME-1 decreased. This confirms our hypothesis that α-syn can regulate PP2A methylation via LCMT-1 and PME-1. The expression of LCMT-1 and PME-1 have recently been found to be post-trancriptionally regulated by microRNA-195 (Liu et al., 2016a). Future work should focus on the mechanism underlying decreased LCMT-1 and increased PME-1 regulation by α-syn. As PP2A inactivation is involved in apoptotic cell death, we investigated and found that LCMT-1 overexpression or PME-1 inhibition reversed α-syn-induced increases in α-syn phosphorylation, and prevented apoptosis. More recently, LCMT-1 Tg mice showed resistance against amyloid β-protein (Aβ) injury, indicating that LCMT-1 or PME-1 can be used as a target for treatment of neurodegenerative diseases (Nicholls et al., 2016). One explanation for this attenuation in apoptosis is the decrease in phosphorylated α-syn. Another mechanism could be that increased PP2A methylation is associated with enhanced autophagy that is contributing to the clearance of excess α-syn. Decreased total α-syn protein levels were observed in the LCMT-1 overexpression and in the AMZ30-treated groups. Methionine and SAM had been shown to regulate cell growth and autophagy via the methylation of PP2A (Sutter et al., 2013). There is also considerable evidence that PP2A inhibition leads to an autophagy deficiency (Du et al., 2015; Liu et al., 2016b).

The regulatory subunits which govern the specificity of phosphatases have recently been getting more attention in neurodegenerative disease and cancer research. They consist of the *PPP2R2*, *PPP2R3*, *PPP2R5*, and *STRN* subfamilies (Perrotti and Neviani, 2013). We observed a reduction in the amount of *PPP2R2* subfamily protein levels in the examined brain regions of α-syn Tg mice. However, *PPP2R5* subfamily protein levels in the same brain regions displayed no significant change. This can be explained by the fact that PP2A methylation is required for its holoenzyme assembly with regulatory subunits of the *PPP2R2* subfamily (Wu et al., 2000). Interestingly, apolipoprotein E ε4 (APOE ε4), which is a genetic risk factor for AD and dementia with Lewy bodies, can cause PP2A inactivation by downregulating the expression of *PPP2R5E*, a regulatory subunit of the *PPP2R5* subfamily (Theendakara et al., 2017; Guerreiro et al., 2018). This suggests that regulatory subunits may play distinct roles at different stages of different diseases, and therefore, more attention should be focused on these subunits. Our data are also consistent with a prior work that showed a reduction in *PPP2R2B* mRNA expression in the midbrain of PD individuals (Kim et al., 2017). Transcriptional regulation may also be involved in this process; this remains undetermined. Folate deficiency-induced tau hyperphosphorylation can be attenuated by *PPP2R2A* overexpression (Sontag et al., 2008). The findings of our study imply that targeting other regulatory subunits belonging to the *PPP2R2* subfamily may be beneficial for treating α-synucleinopathies. Additionally, there are other endogenous regulators, that include SET and PTPA, which may contribute to PP2A inhibition in neurodegenerative diseases (Arnaud et al., 2011; Zhang et al., 2014). Future work should focus on the mechanisms underlying α-syn regulation of these genes’ expression.

## conclusion

**FIGURE 7:**
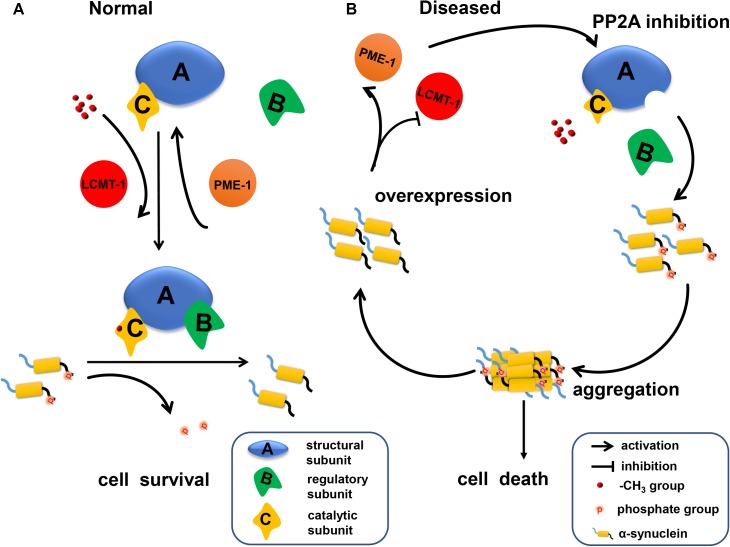
A schematic representation indicating that α-syn inhibited PP2A activity via downregulating LCMT-1 expression and upregulating PME-1 expression. **(A)** In normal conditions, phosphorylated α-syn at Ser129 is a substrate of PP2A holoenzyme under the methylation state. PP2A methylation is regulated by LCMT-1 and PME-1. **(B)** In the state of α-syn overexpression, PP2A inhibition was mediated by decreased PP2A methylation and expression of specific subfamily regulatory subunits. This inhibition promoted α-syn hyperphosphorylation at Ser129 and aggregation. Consequently, neurodegeneration and cell death occurred.

Our data indicates that α-syn increases PP2A demethylation via downregulation of LCMT-1 expression, and upregulation of PME-1 expression. This process is accompanied by decreased *PPP2R2* subfamily regulatory subunit expression, resulting in an eventual decrease in PP2A activity toward α-syn (**Figure 7**). Under these circumstances, phosphorylated α-syn, which can further inhibit PP2A activity, significantly increases. Therefore, there is a positive feedback loop between α-syn and PP2A. Our work provides new targets for PD treatment.

## Author Contributions

HT and HY conceived and designed the study and wrote the manuscript. HT, JL, YL, WL, LL, CD, GG, and HY performed all of the experiments and analyzed data.

## Conflict of Interest Statement

The authors declare that the research was conducted in the absence of any commercial or financial relationships that could be construed as a potential conflict of interest.
