# Supplementary material for: Leucine Carboxyl Methyltransferase Downregulation and Protein Phosphatase Methylesterase Upregulation Contribute Toward the Inhibition of Protein Phosphatase 2A by α-Synuclein
Source: Front Aging Neurosci. 2018 Jun 8;10:173. doi: 10.3389/fnagi.2018.00173 (PMC6008559; doi:10.3389/fnagi.2018.00173)
Supplement: Supplementary file 1 [file Data_Sheet_1.DOCX]

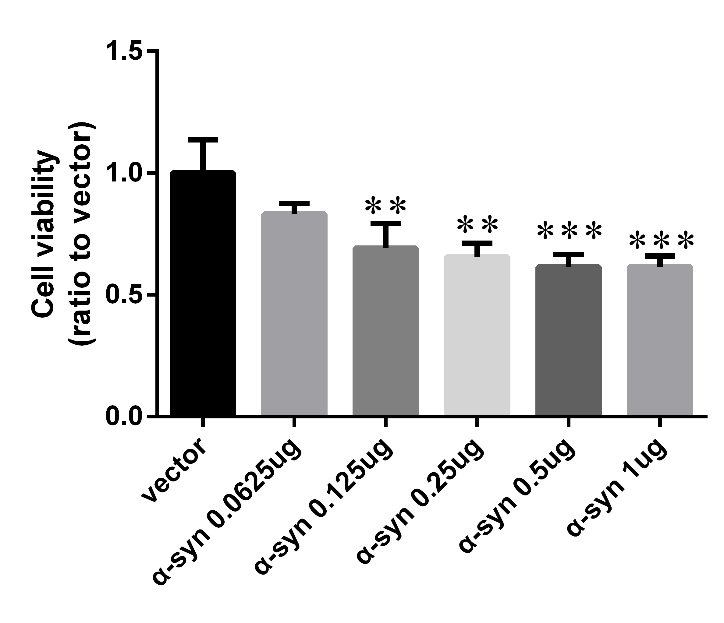


**Supplementary Figure 1. α-syn induced a dose-dependent decrease in cell viability after transfection for 24 h.**

Cell viability was measured in SK-N-SH cells transfected with increasing amounts of pCMV-Myc-α-syn (0.0625, 0.125, 0.25, 0.5 and 1 µg). pCMV-Myc plasmid was added to keep the total amount of DNA equal in each transfection. Data are expressed as mean ± SD. Brown-Forsythe test; P>0.05; one-way analysis of variance; **P<0.01, ***P<0.001 vs. vector (n=3).





**Supplementary Figure 2. PP2Ac overexpression induced increases in PP2A activity while its 309A mutant (PP2Ac 309A) abolished this increase.** Measurement of PP2A activity in SK-N-SH cells transfected with PP2A catalytic subunit (PP2Ac) or 309A mutant (PP2Ac 309A) which can not be methylated. Data are expressed as mean ± SEM. Brown-Forsythe test; P>0.05; one-way analysis of variance; *P<0.05 vs. vector (n=3); ^#^P<0.05 vs. PP2Ac (n=3).


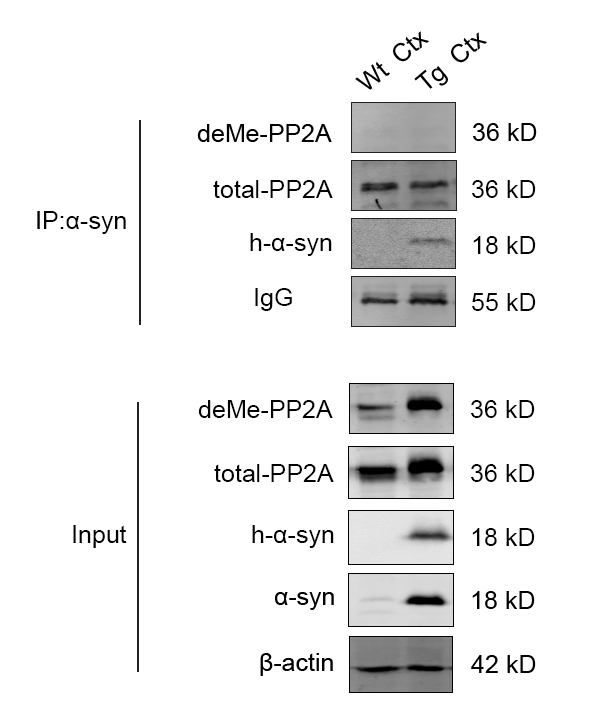


**Supplementary Figure 3. α-syn did not interact with demethylated PP2A.**

Interaction between α-syn and PP2A was detected by Co-IP in the cortex of Tg mice and Wt littermates.


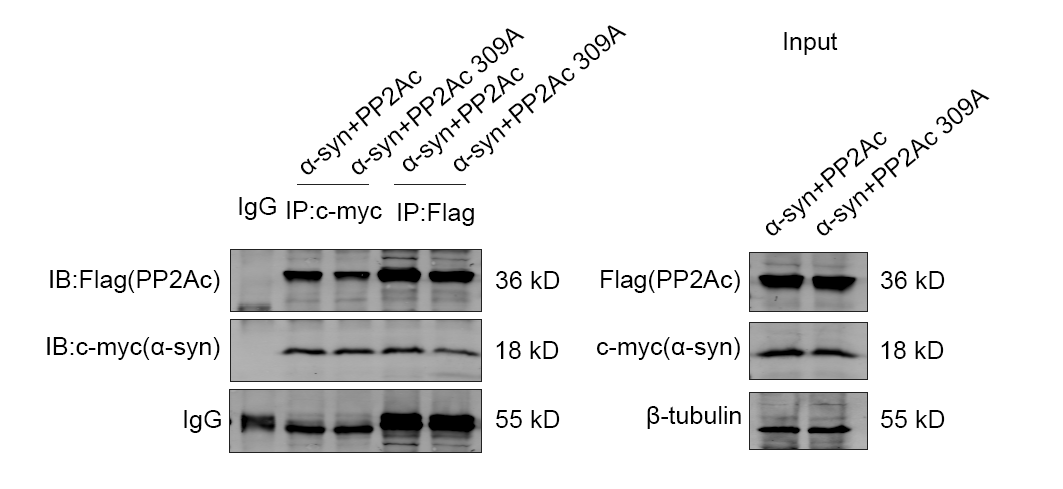


**Supplementary Figure 4. Compared with wild type PP2Ac, the interaction between α-syn and PP2Ac 309A significantly decreased.** SK-N-SH cells were transfected with myc-α-syn and flag-PP2Ac or 309A mutant; Interaction between PP2Ac and α-syn was detected by Co-IP.


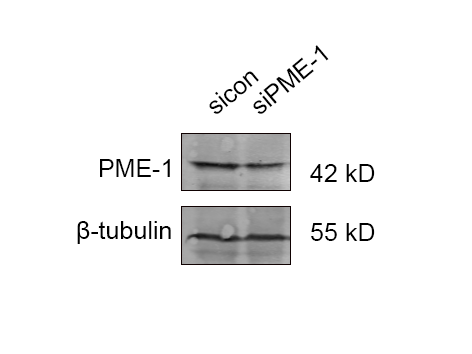

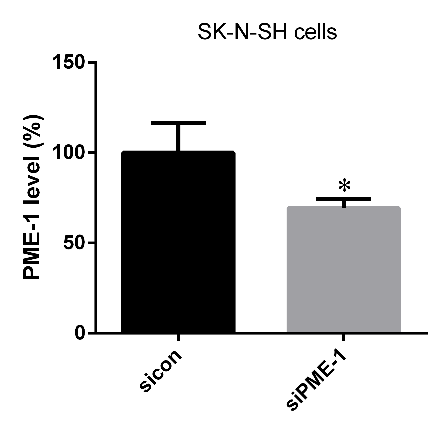


**Supplementary Figure 5. Transfection efficiency of PME-1 siRNA was assessed.**

Western blot results and quantitative analysis of PME-1 in SK-N-SH cells transfected with scramble siRNA (sicon) or PME-1 siRNA (siPME-1). The ratio of PME-1 expression to β-tubulin in scramble siRNA group was considered as 100%. Data are expressed as mean ± SD. F-test; P>0.05; unpaired Student’s t-test; *P<0.05, vs. scramble siRNA group (n=3).


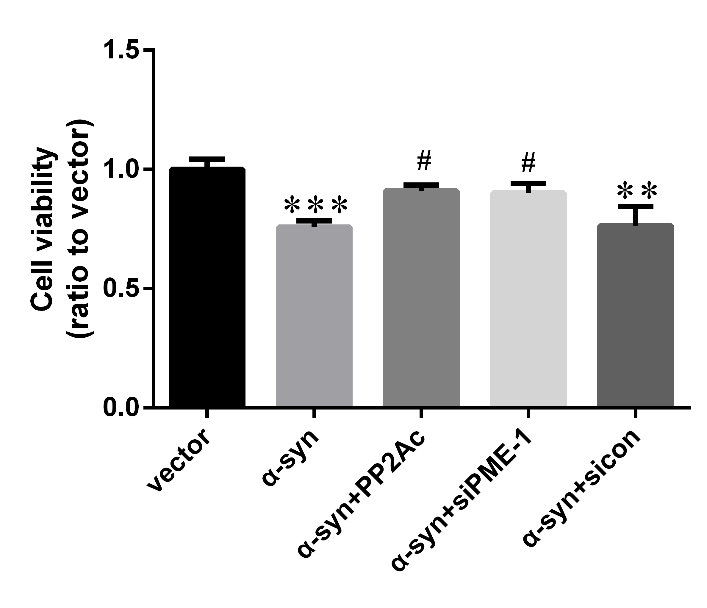


**Supplementary Figure 6. Catalytic subunit of protein phosphatase 2A (PP2Ac) overexpression or PME-1 knockdown protected against cell apoptosis mediated by α-syn.**

Cell viability was measured in vector, α-syn, α-syn + PP2Ac, α-syn + siPME-1 and α-syn + sicon group. Data are expressed as mean ± SD. Brown-Forsythe test; P>0.05; one-way analysis of variance; **P<0.01, ***P<0.001 vs. vector (n=3); ^#^P<0.05 vs. α-syn (n=3).


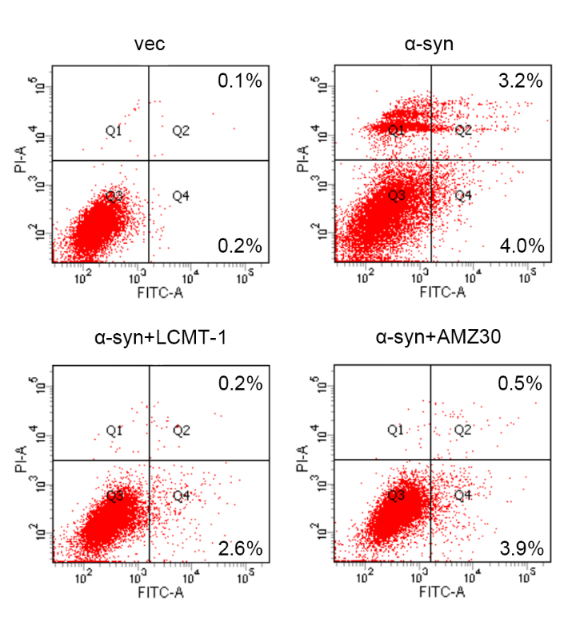

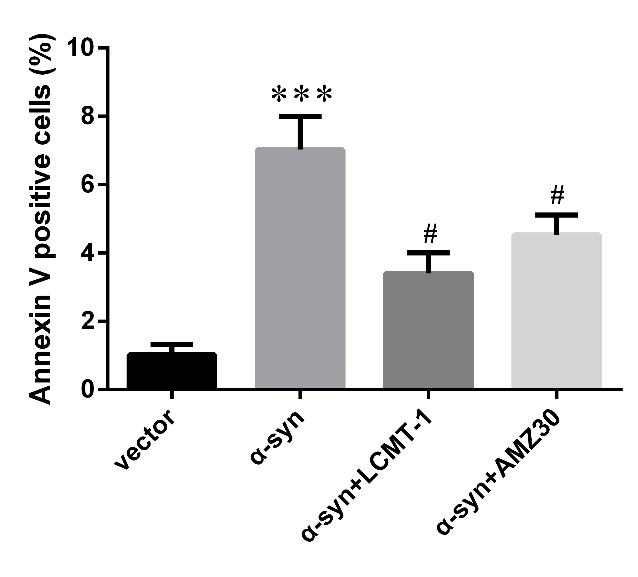


**Supplementary Figure 7. LCMT-1 overexpression or PME-1 inhibition by AMZ30 protected against cell apoptosis mediated by α-syn.**

Cell apoptosis was assessed by flow cytometry analysis. Data are expressed as mean ± SEM. Brown-Forsythe test; P>0.05; one-way analysis of variance; ***P<0.001 vs. vector (n=3); ^#^P<0.05 vs. α-syn (n=3).
